# Isoliquiritigenin Protects Against Pancreatic Injury and Intestinal Dysfunction After Severe Acute Pancreatitis via Nrf2 Signaling

**DOI:** 10.3389/fphar.2018.00936

**Published:** 2018-08-17

**Authors:** Man Zhang, Yan-Qing Wu, Ling Xie, Jiang Wu, Ke Xu, Jian Xiao, Da-Qing Chen

**Affiliations:** ^1^Department of Emergency, The Second Affiliated Hospital and Yuying Children’s Hospital of Wenzhou Medical University, Wenzhou Medical University, Wenzhou, China; ^2^Molecular Pharmacology Research Center, School of Pharmaceutical Science, Wenzhou Medical University, Wenzhou, China; ^3^Wenzhou University College of Life and Environmental Science, Wenzhou, China

**Keywords:** severe acute pancreatitis, intestinal dysfunction, isoliquiritigenin, Nrf2^-/-^, NF-κB, oxidative stress

## Abstract

Severe acute pancreatitis (SAP) is a digestive system disease that is associated with a range of complications including intestinal dysfunction. In this study, we determined that the chalcone compound, isoliquiritigenin (ISL), reduces pancreatic and intestinal injury in a mouse model of SAP. These effects were achieved by suppressing oxidative stress and the inflammatory responses to SAP. This was evidenced by a reduction in histological score, and malondialdehyde (MDA), interleukin (IL)-6, tumor necrosis factor (TNF)-α and cleaved-caspase-3 (c-caspase-3) protein along with an increase in Nrf2, hemeoxygenase-1 (HO-1), quinone oxidoreductase 1 (NQO1), and superoxide dismutase (SOD). We then used Nrf2^-/-^ mice to test the protective effect of Nrf2 during ISL treatment of SAP. Our results indicated that Nrf2^-/-^ mice had greater pancreatic injury and intestinal dysfunction than wild-type mice. They also had reduced adherens junctions (P120-catenin) and tight junctions (occludin), and increased activated nuclear factor-κB (NF-κB) protein. In Nrf2^-/-^ mice, ISL was less effective at these functions than in the WT mice. In conclusion, this study demonstrated that ISL exerts its protective effects against oxidative stress and inflammatory injury after SAP via regulation of the Nrf2/NF-κB pathway. It also showed that the efficacy of ISL in repairing the intestinal barrier damage caused by SAP is closely related to the Nrf2 protein. Our findings demonstrated that Nrf2 is an important protective factor against SAP-induced injuries in the pancreas and intestines.

## Introduction

Severe acute pancreatitis (SAP) is a clinical emergency that causes considerable morbidity and carries a high mortality. The underlying etiology of SAP has not been fully elucidated, but it is clear that it produces damage through the complex pathological process itself and also its complications (Zerem, 2014; Xiang et al., 2017). The disease process involves oxidative stress (Deng et al., 2016), autophagy (Gukovsky et al., 2013; Ji et al., 2016) and an inflammatory response (Nagao et al., 2016; He et al., 2017), whereas the many complications include acute renal failure, heart failure and arrhythmia, and gastrointestinal bleeding and dysfunction (Kambhampati et al., 2014; Karakayali, 2014; Xiang et al., 2017). While there have been advances in the diagnosis and treatment of SAP, current approaches to managing this disease are inadequate and mortality remains high. To date, there is no effective treatment for SAP.

The inflammatory and oxidative stress responses to pancreatitis are important contributors to disease progression (Cikman et al., 2015; Xiong et al., 2018). Studies in mice have shown that these responses aggravate pancreatitis-induced injury (Park et al., 2014; Gooshe et al., 2015). Thus, inhibition of the inflammatory and oxidative stress responses may assist in the treatment of SAP. NF-κB and Nrf2 play important roles in the inflammatory and oxidative stress response, with some studies suggesting that these proteins may contribute to pancreatitis development (Genrich et al., 2016; Liu Y. et al., 2017). Thus, regulating the expression of NF-κB and Nrf2 proteins may improve the prognosis of this disease. Cell and mouse studies have shown that the inflammatory response can be suppressed through the Nrf2/NF-κB pathway (Jin et al., 2008a; Pan et al., 2011). However, whether Nrf2 exerts its effect on NF-κB activity in SAP is unclear. Another potential therapeutic intervention is to prevent SAP complications such as destruction of intestinal function (Xiong et al., 2018). This induces translocation of intestinal bacteria and endotoxin, which leads to the systemic inflammatory response syndrome and multiple organ failure (Zhang et al., 2015). Therefore, mitigation of bowel dysfunction may reduce the damage caused by SAP. Previous studies have shown that the damaged intestinal barrier is repaired via the Nrf2/ARE signaling pathway (Hu et al., 2018) and that Nrf2 regulates airway epithelial barrier integrity (Chen et al., 2014). There have been no published reports investigating whether Nrf2 can repair the damaged intestinal barrier caused by SAP.

Isoliquiritigenin (ISL) is a chalcone compound that has many pharmacological effects. Earlier studies have shown that ISL suppresses the inflammatory and oxidative stress response in mice (Du et al., 2016; Zeng et al., 2017). It also protects against sepsis-induced lung and liver injury via its anti-inflammatory actions (Chen et al., 2017). These results suggest that ISL may be a candidate drug when antagonistic effects are needed against inflammation and oxidative stress. To date, there have been no studies published investigating whether ISL has anti-inflammatory and anti-oxidant effects in SAP-associated intestinal dysfunction. Prior work suggests that ISL could repair barrier function in the brain injury model (Zeng et al., 2017), signifying that it could assist in SAP-induced intestinal barrier dysfunction. It has been reported that the blood–brain barrier in mice can be repaired by regulating the Nrf2 pathway (Hoyles et al., 2018), and that Nrf2 expression increases in intestinal dysfunction (Trivedi et al., 2016). In addition, studies evaluating lung injury and cerebral ischemia have shown that NF-κB and Nrf2 proteins can be regulated by ISL (Liu Q. et al., 2017; Zeng et al., 2017). It has also been reported that NF-κB activity is inhibited by ISL through activation of the Nrf2 signal pathway. However, it is not known whether ISL exerts an effect in SAP via regulation of NF-κB and Nrf2 proteins. Collectively, these studies have led us to question whether ISL can inhibit inflammation and the oxidative stress response as well as repair intestinal barrier function, and whether this occurs via regulation of the Nrf2 protein in the SAP model.

In this study, we investigated the effect of ISL and Nrf2 protein in SAP-associated intestinal dysfunction. In the SAP model, we found that ISL reduced the inflammatory and oxidative stress response in the pancreas and the intestines via the Nrf2/NF-κB pathway. Furthermore, we demonstrated that mice deficient in Nrf2 were more susceptible to injury in SAP and that the Nrf2 protein is a protective factor against SAP-induced destruction of the intestinal barrier.

## Materials and Methods

### Animals and Model

Wild-type (WT) C57BL/6 male mice (20–25 g) were obtained from the Animal Center of Wenzhou Medical University (Wenzhou, China). Nrf2^-/-^ mice on C57BL background were obtained from the Experiment Animal Centre of Nanjing Medical University (Jiangsu, China). All experiments were conducted with the approval of the Animal Care and Use Committee of Wenzhou Medical University (Wenzhou, China). The animals were maintained on a 12:12 hour (h) light–dark cycle with free access to water and food for at least 1 week before any experimentation. All experiments were conducted in the morning. The mice were fasted for 12 h prior to experimentation. The SAP model was induced by intraperitoneal injections of cerulein 50 μg/kg administered in sodium chloride (0.9%) each hour for seven doses. Lipopolysaccharide (LPS) 10 mg/kg was added to the last cerulein injection. The mice were killed 24 h after the first cerulein injection.

### Reagents and Chemicals

Isoliquiritigenin was obtained from the Aladdin Company (Shanghai, China). Cerulein and LPS were purchased from Sigma Chemical (Sigma-Aldrich, St. Louis, MO, United States). Antibodies against cleaved caspase-3 (c-caspase-3) and NF-κB p65 were from Cell Signaling Technology (CST, Danvers, MA, United States). Antibodies against Nrf2, occludin, and p-120catenin were from Abcam (Cambridge, MA, United States). Anti-GAPDH antibody, anti-mouse secondary antibodies and anti-rabbit secondary antibodies were purchased from MultiSciences Biotech, Co. (Hangzhou, China). IL-6 and TNF-α enzyme-linked immunosorbent assay (ELISA) kits were purchased from eBioscience (San Diego, CA, United States).

### Western Blot Analysis

The samples were subjected to 10% SDS-PAGE and transferred onto a PVDF membrane (Bio-Rad Laboratories, Hercules, CA, United States). The membranes were blocked with 5% fat-free milk at room temperature for 2 h and then incubated with the following primary antibodies: anti-GAPDH (1:10000), anti-NF-κB (1:1000), anti-Nrf2 (1:1000), anti-occludin (1:1000), anti-p-120catenin (1:1000), and anti-cleaved caspase-3 (1:1000) at 4°C overnight. The membranes were then washed with TBST and incubated with a secondary horseradish peroxidase-conjugated antibody at room temperature for 1 h. The signals were visualized with the ChemiDicTM XRS+ Imaging System (BioRad Laboratories, Hercules, CA, United States), and the densities of the immunoreactive bands were analyzed using ImageJ software (NIH, Bethesda, MD, United States).

### Histological Examination and Immunofluorescence Assay

The mice were anesthetized and then perfused successively with sodium chloride 0.9 and 4% (w/v) paraformaldehyde (PFA). The pancreas and intestinal tissue were collected and embedded in paraffin wax after fixing in 4% PFA for 24 h. Sections (5 μm thick) were then mounted onto poly L-lysine-coated slides and stained with hematoxylin for histopathological examination. Images were captured by a Nikon ECLPSE 80i (Nikon, A1 PLUS, Tokyo, Japan). Intestinal histological damage was scored from 0 to 5 as described previously (Deng et al., 2016). In brief: grade 0, normal mucosal villi; grade 1, development of subepithelial Gruenhagen’s space at the apex of the villus; grade 2, extension of the subepithelial space with moderate lifting of the epithelial layer from the lamina propria; grade 3, massive epithelial lifting down the sides of villi, possibly with a few denuded tips; grade 4, denuded villi with the lamina propria and dilated capillaries exposed; and grade 5, digestion and disintegration of the lamina propria, hemorrhage, and ulceration. The pancreatic histological scores were also based on a method described previously (Schmidt et al., 2015). Briefly, these comprised four main criteria: edema, acinar necrosis, hemorrhage and fat necrosis, and inflammation and infiltration. The scoring assessment was performed on a scale of 0 (normal) – 4 (severe) for each criterion with the sum of the scores used to evaluate severity of the acute pancreatitis. For the immunofluorescence assay, the sections were treated with 5% bovine serum albumin (BSA) in PBS for 30 min after deparaffinization and rehydration. The samples were then stained with the specific primary antibodies: anti-NF-κB (1:500), anti-Nrf2 (1:300), anti-occludin (1:200), anti-p-120catenin (1:300), and anti-cleaved caspase-3 (1:500) at 4°C overnight. After washing with PBS, the samples were incubated with the secondary antibody (1:1000) at 37°C for 1 h. The sections were then restained with 4′-6-diamidino-2-phenylindole (DAPI) for 7 min. The fluorescent images were captured by a Nikon confocal laser microscope (Nikon, A1PLUS, Tokyo, Japan).

### Real-Time Quantitative Polymerase Chain Reaction (RT-qPCR)

Total RNA was extracted from the tissues using TRIzol (Invitrogen, Carlsbad, CA, United States). The Prime Script RT-PCR kit (TaKaRa Bio, Dalian, China) was used for reverse transcription as per the manufacturer’s instructions. The concentration of total RNA was detected by nucleic acid protein analyzer (Beckman Coulter, Inc., Brea, CA, United States). Real-time qPCR was amplified with the Eppendorf Real Plex 4 instrument (Eppendorf, Hamburg, Germany) and the specific sequences of the primers (Invitrogen Shanghai, China) were as follows: mouse, TNF-α forward: TGATCCGCGACGTGGAA, reverse: ACCGCCTGGAGTTCTGGAA; IL-6 forward: CCAAGAGGTGAGTGCTTCCC, reverse: CTGTTGTTCAGACTCTCTCCCT; β-actin forward: CCGTGAAAAGATGACCCAGA, reverse: TACGACCAGAGGCATACAG, NQO1 forward: CAT TCT GAA AGG CTG GTT TGA, reverse: CTA GCT TTG ATC TGG TTG TCAG; HO-1 forward: ATCGTGCTCGCATGAACACT, reverse: CCAACACTGCATTTACATGGC.

### Determination of Malondialdehyde (MDA) and Superoxide Dismutase (SOD) Concentrations

The concentrations of MDA and SOD were detected using the appropriate kits as per the manufacturer’s instructions (Beyotime Biotech, Inc., Jiangsu, China). The pancreatic and intestinal tissue were homogenized and centrifuged at 12000 *g* for 15 min before collecting the supernatant to investigate spectrophotometrically.

### Statistical Analysis

The data are presented as the mean ± standard error of the mean (SEM). Statistical analyses were determined by one-way analysis of variance (ANOVA) using GraphPad Pro 5.0 software (San Diego, CA, United States). *P*-values < 0.05 were considered significant. All experiments were carried out at least three times.

## Results

### Nrf2 Depletion Aggravates SAP-Induced Damage and Attenuates the Effect of ISL Inhibition on Pancreatic Damage

**FIGURE 1:**
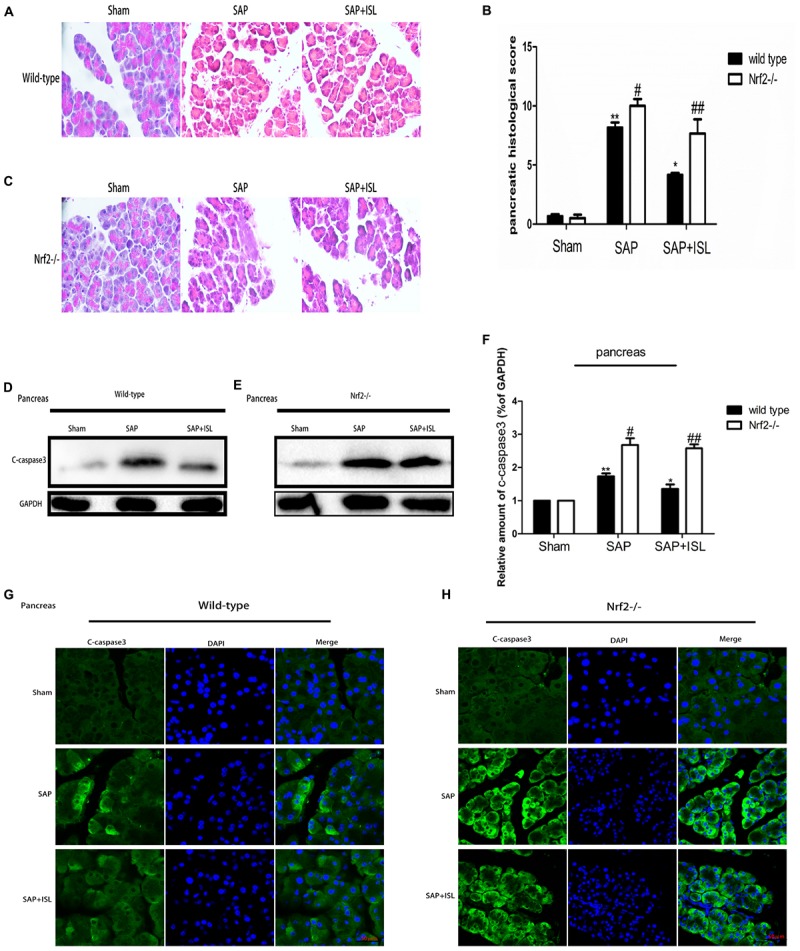
Isoliquiritigenin (ISL) treatment protects against combined cerulein plus LPS-induced severe acute pancreatitis (SAP) in pancreatic tissue. **(A,C)** Pancreatic morphological changes in the different groups at 24 h after SAP. **(B)** Pancreatic histological scores. **(D,E)** Expression of c-caspase-3 protein in pancreatic tissue from the different groups at 24 h after SAP. GAPDH was used as the loading control and for band density normalization. **(F)** Statistical graph of c-caspase-3 proteins. **(G,H)** Immunofluorescence staining for c-caspase-3 (green) and DAPI (blue) in the different groups. Results are expressed as mean ± SEM. *n* = 5 per group. ^∗^P< 0.05 and ^∗∗^P < 0.01 when comparison was made in WT mice. ^#^ P < 0.05 and ^##^ P < 0.01 when comparison was made between WT mice and Nrf2^-/-^ mice.

We evaluated the therapeutic role of ISL in SAP-associated pancreatic injury by assessing the pathological changes. The HE staining assay showed that edema and leukocyte infiltration was present in the SAP group but not in the sham group, whereas the ISL group had normal pathological features and lower pathological scores than the SAP group (*P* < 0.05) (**Figures 1A,B**). As apoptosis is another important factor of SAP-induced injury we investigated the activity of caspase-3 in pancreatic tissue. We found the expression of c-caspase-3 proteins to be elevated in pancreatitis, and ISL treatment to mitigate this (**Figure 1D**). Compared with WT mice, administration of cerulein with LPS to Nrf2^-/-^ mice caused more severe pathological changes in the pancreatic tissue, with a high histopathological score including obvious acinar cell vacuolization and necrosis, and edema (**Figures 1B,C**). Furthermore, concentrations of the cleavage product of caspase-3 significantly increased in Nrf2^-/-^ mice compared with WT mice (**Figures 1E,F**). ISL treatment could not reverse the trend observed in the Nrf2^-/-^ mice compared with WT mice (**Figures 1E–H**).

### Nrf2 Depletion Aggravates SAP-Induced Damage and Attenuates the Effect of ISL Inhibition on Intestinal Tissue Damage

**FIGURE 2:**
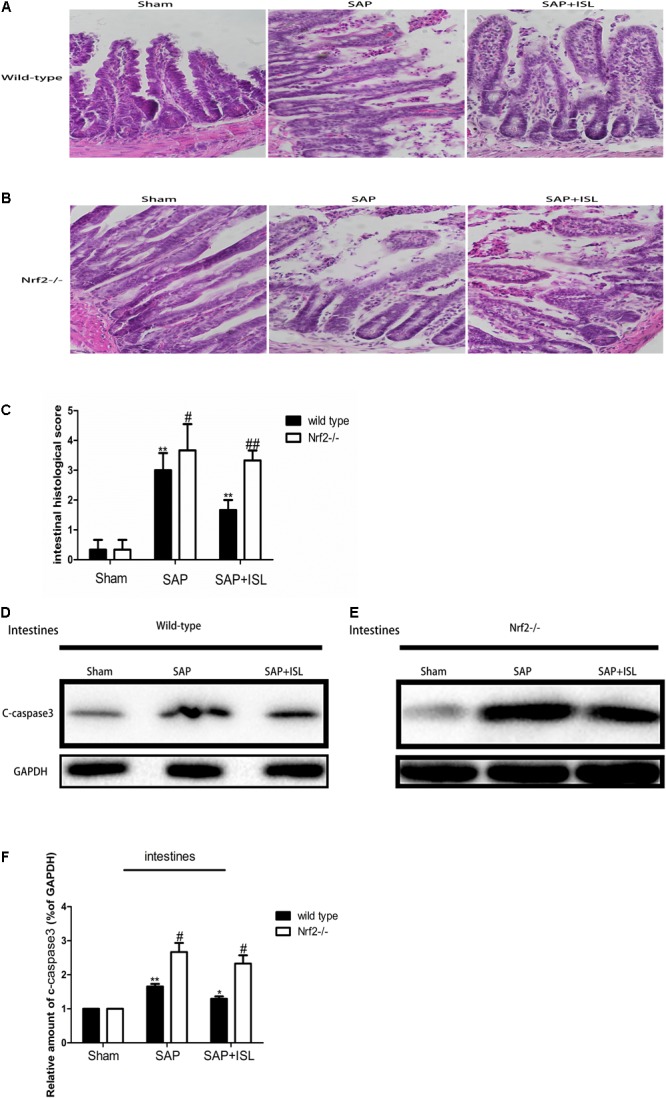
Isoliquiritigenin treatment protects against combined cerulein plus LPS-induced SAP in intestinal tissue. **(A,B)** Intestinal morphological changes of the different groups at 24 h after SAP. **(C)** Intestinal histological scores. **(D,E)** Protein expression of c-caspase-3 in intestinal tissue from the different groups at 24 h after SAP. GAPDH was used as the loading control and for band density normalization. **(F)** Statistical graph of c-caspase-3 proteins. Results are expressed as mean ± SEM. *n* = 5 per group. ^∗^ P < 0.05 and ^∗∗^ P < < 0.01 when comparison was made in WT mice. ^#^ P < 0.05 and ^##^ P < 0.01 when comparison was made between WT mice and Nrf2^-/-^ mice.

To test the effect of Nrf2 on intestinal tissue after SAP we measured the pathological changes in intestinal tissue. We found that acinar cell necrosis, interstitial edema and inflammatory cell infiltration occurred in the SAP group, and ISL treatment obviously lessened these changes (**Figure 2A**). The expression of c-caspase-3 proteins was also elevated in intestinal tissue, and ISL treatment mitigated this (**Figure 2D**). Consistently, the morphological intestinal changes were also more severe in Nrf2^-/-^ mice than in WT mice. These changes included exfoliated and incomplete intestinal mucosa, edema and infiltration of inflammatory cells, loss of goblet cells and red blood cell effusion (**Figures 2B,C**). In WT mice, low levels of the cleavage product of caspase-3 were detected after cerulein plus LPS treatment. In contrast, these were significantly increased in the Nrf2^-/-^ mice compared with WT mice (**Figures 2E,F**). ISL was less effective in Nrf2^-/-^ mice compared with WT mice (**Figures 2D–F**).

### Nrf2 Depletion Elevated SAP-Induced Inflammatory and Oxidative Stress Responses, and Weakened the Efficacy of ISL at Suppressing These

**FIGURE 3:**
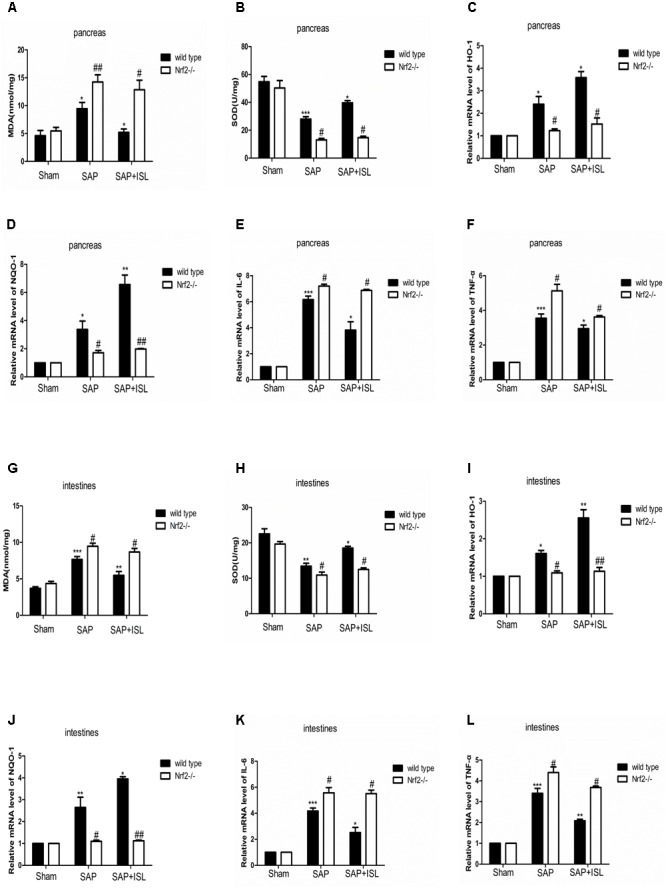
Isoliquiritigenin treatment reduces inflammation and the oxidative stress response in WT and Nrf2^-/-^ mice after SAP. **(A,B)** Concentration of MDA and SOD in pancreatic tissue from the different groups. **(C,D)** HO-1 and NQO-1 mRNA levels in pancreatic tissue were analyzed by real-time qPCR in the different groups using β-actin mRNA as the internal control. **(E,F)** IL-6 and TNF-α mRNA levels were analyzed in pancreatic tissue by real-time qPCR in the different groups using β-actin mRNA as the internal control. **(G,H)** Concentration of MDA and SOD in the intestinal tissue of the different groups. **(I,J)** HO-1 and NQO-1 mRNA levels were analyzed in intestinal tissue by real-time qPCR in the different groups using β-actin mRNA as the internal control. **(K,L)** IL-6 and TNF-α mRNA levels were analyzed in intestinal tissue by real-time qPCR in the different groups using β-actin mRNA as the internal control. Results are expressed as mean ± SEM. *n* = 5 per group. ^∗^ P < 0.05, ^∗∗^ P < 0.01, and ^∗∗∗^ P < 0.001 when comparison was made in WT mice. ^#^ P < 0.05 and ^##^ P < 0.01 when comparison was made between WT mice and Nrf2^-/-^ mice.

To investigate whether ISL slowed the inflammatory and oxidative stress response after SAP we measured inflammatory factors, IL-6 and TNF-α, and oxidative stress-related indicators, MDA (a marker of membrane lipid peroxidation) and SOD (anti-oxidant enzymes responsible for scavenging metabolites produced by free-radicals) in pancreatic and intestinal tissue. In WT mice, pancreatic and intestinal IL-6 and TNF-α were significantly elevated after SAP, and ISL attenuated these changes (**Figures 3E,F,K,L**). Tissue concentrations of MDA were higher in the SAP group than the sham group, and were significantly decreased with ISL compared with the SAP group (**Figures 3A,G**). SOD was significantly less in the SAP group compared with the sham group, and antioxidant enzyme concentrations were higher in the ISL group than in the SAP group (**Figures 3B,H**). Furthermore, ISL increased the expression of Nrf2 downstream cytoprotective proteins HO-1 and NQO-1, which was different with SAP group and sham group (**Figures 3C,D,I,J**). In Nrf2^-/-^mice, we found the concentrations of IL-6 and TNF-α to be significantly increased after combined cerulein and LPS treatment compared with WT mice. ISL treatment was less effective at reducing these effects in Nrf2^-/-^ mice compared with WT mice (**Figures 3E,F,K,L**). Nrf2^-/-^ mice also exhibited higher MDA concentrations and lower SOD, HO-1 and NQO-1 after cerulein plus LPS. ISL treatment also proved less effective in this Nrf2^-/-^ versus WT mice (**Figures 3A–D,G–J**).

### Nrf2 Depletion Elevated NF-κB and Reduced IκB Protein Expression, and Altered ISL Effects on NF-κB and IκB Activity in Pancreatic and Intestinal Tissue After SAP

**FIGURE 4:**
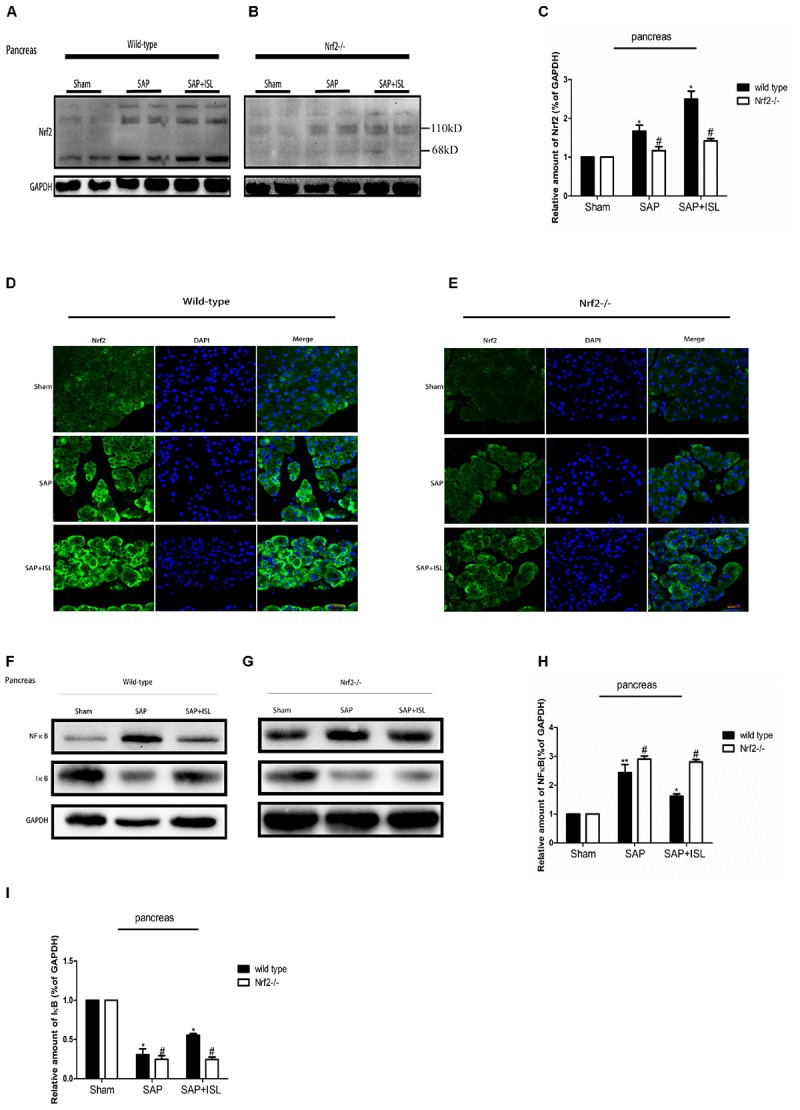
Effect of ISL treatment on NF-κB and Nrf2 in pancreatic tissue in WT and Nrf2^-/-^ mice after SAP. **(A,B)** Protein expression of Nrf2 in pancreatic tissue of the different groups after SAP. GAPDH was used as the loading control and for band density normalization. **(C)** Statistical graph of Nrf2 and GAPDH protein in the different groups. **(D,E)** Immunofluorescence staining for Nrf2 (green) and DAPI (blue) in the different groups. **(F,G)** Protein expression of NF-κB and IκB in pancreatic tissue in the different groups after SAP. GAPDH was used as the loading control and for band density normalization. **(H,I)** Statistical graph of NF-κB, IκB, and GAPDH protein in the different groups. Results are expressed as mean ± SEM. *n* = 5 per group. ^∗^ P < 0.05, ^∗∗^ P < 0.01, and ^∗∗∗^ P < 0.001 when comparison was made in WT mice. ^#^ P < 0.05 when comparison was made between WT mice and Nrf2^-/-^ mice.

**FIGURE 5:**
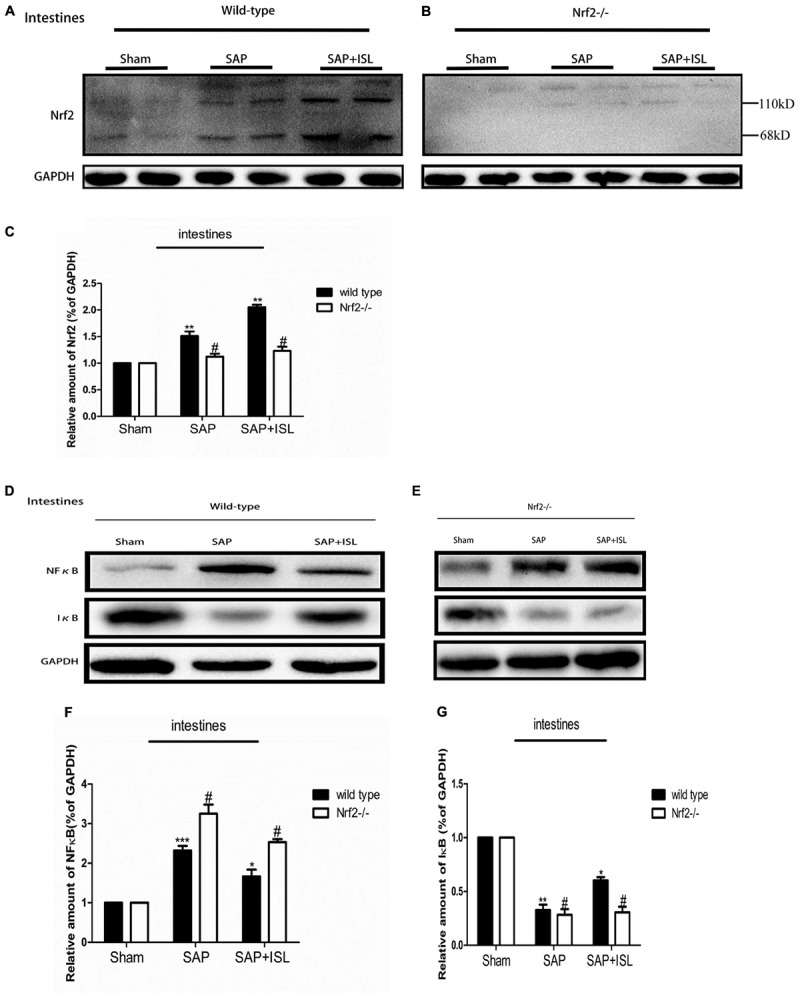
Effect of ISL treatment on NF-κB and Nrf2 in intestinal tissue in WT and Nrf2^-/-^ mice after SAP. **(A,B)** Protein expression of Nrf2 in intestinal tissue in the different groups after SAP. GAPDH was used as the loading control and for band density normalization. **(C)** Statistical graph of Nrf2 and GAPDH protein in the different groups. **(D,E)** Protein expression of NF-κB and IκB in pancreatic tissue in the different groups after SAP. GAPDH was used as the loading control and for band density normalization. **(F,G)** Statistical graph of NF-κB and IκB. GAPDH protein in the different groups. Results are expressed as mean ± SEM. *n* = 5 per group. ^∗^ P < 0.05, ^∗∗^ P < 0.01, and ^∗∗∗^ P < 0.001 when comparison was made in WT mice. ^#^ P < 0.05 when comparison was made between WT mice and Nrf2^-/-^ mice.

Since ISL can inhibit oxidative stress and the inflammatory response, we evaluated NF-κB and IκB activation, and Nrf2 expression in pancreatic and intestinal tissue. In WT mice, western blot analysis showed that the expression of NF-κB was elevated in the SAP group compared with the sham group, while the ISL treatment group had reduced expression of NF-κB protein compared with the SAP group (**Figures 4F, 5D**). IκB protein was significantly less in the SAP group compared with the sham group. The ISL treatment group also had higher IκB protein than the SAP group (**Figures 4F, 5D**), while Nrf2 was increased, which was different to the sham and SAP groups (**Figures 4A,D, 5A**). To investigate the impact of Nrf2 deletion on the effect of ISL against the inflammatory response to SAP, we built the SAP model in Nrf2^-/-^ mice. Following cerulein plus LPS-induced SAP, we found the expression of NF-κB to be high, Nrf2 and IκB to below in Nrf2^-/-^ mice compared with WT mice (**Figures 4B,C,E–J, 5B–G**). Further, ISL treatment of cerulein plus LPS-induced SAP was less effective in Nrf2^-/-^ mice compared with WT mice (**Figures 4B,C,E–J, 5B–G**).

### Nrf2 Depletion Damaged Intestinal Barrier Integrity and Weakened the Effect of ISL in Maintaining Intestinal Integrity After SAP

**FIGURE 6:**
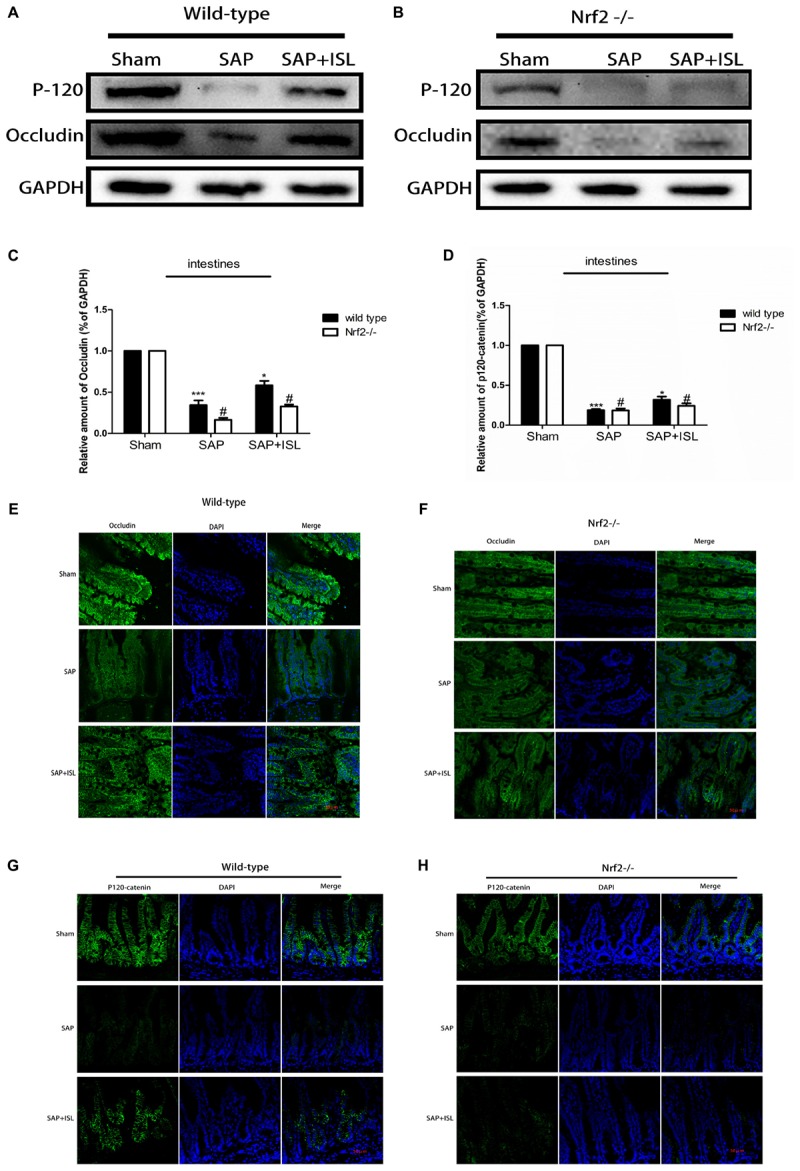
Effect of Nrf2 deletion on maintenance of intestinal barrier integrity after SAP. **(A,B)** Protein expression of P-120-catenin and occludin in pancreatic and intestinal tissue in the different groups after SAP. GAPDH was used as the loading control and for band density normalization. **(C,D)** Statistical graph of P-120-catenin, occludin, and GAPDH protein in the different groups. **(E–H)** Immunofluorescence staining for P-120-catenin (green), occludin (green), and DAPI (blue) in the different groups. Results are expressed as mean ± SEM. *n* = 5 per group. ^∗^ P < 0.05 and ^∗∗∗^ P < 0.001 when comparison was made in WT mice. ^#^ P < 0.05 when comparison was made between WT mice and Nrf2^-/-^ mice.

As a breakdown in the intestinal barrier results in intestinal dysfunction we assessed the adherens junctions (AJs) and tight junctions (TJs) via western blot analysis of the expression of P120-catenin and occludin proteins. In WT mice, these proteins were both reduced in the SAP group versus the sham group, while the ISL treatment group had elevated expression of both proteins compared with the SAP group (**Figure 6A**). In addition, the expression of P120-catenin and occludin proteins were both at low levels in the Nrf2^-/-^ mice and were significantly lower than in the WT mice (**Figures 6B–D**). Correspondingly, immunostaining of P120-catenin and occludin illustrated that Nrf2 deletion reduces their expression in SAP (**Figures 6F,H**). To further study the effects of Nrf2 deletion on maintenance of intestinal barrier integrity in SAP, we assessed the expression of intestinal P120-catenin and occludin after ISL treatment of SAP. We found that the expression of these proteins was low in Nrf2^-/-^ mice after treatment with ISL in SAP. In contrast, ISL treatment of cerulein plus LPS-induced SAP increased the expression of these proteins in WT mice compared with Nrf2^-/-^mice (**Figures 6E–H**).

## Discussion

Severe acute pancreatitis is a serious disease with complex underlying mechanisms that are poorly understood. As current treatment strategies are inadequate, SAP causes considerable morbidity and mortality. Clinical practice and research studies indicate that the pathological processes of pancreatitis and its complications are the two major factors underlying SAP-associated morbidity and mortality (Zerem, 2014; Shah et al., 2018). The two most common disease processes are inflammation (Bonior et al., 2017) and oxidative stress (Deng et al., 2016), whereas intestinal dysfunction is a frequent extra-pancreatic complication (Zhang et al., 2015). Effective treatment options are lacking resulting in patient suffering and high treatment costs. In this context, it is necessary to find an effective therapy for the management of SAP. ISL has been shown to have a significant beneficial effect in lung (Liu Q. et al., 2017) and liver injury (Chen et al., 2017), and in cerebral ischemia (Zeng et al., 2017) via antagonistic effects against inflammation and oxidative stress. In contrast with other bodily systems, the gastrointestinal system contains many digestive enzymes that can inactivate pharmacological therapies rendering them less effective. Thus, in this study, we investigated the protective effects of ISL in pancreatic and intestinal tissue using a mouse model of SAP. We found that ISL protects against pancreatic injury and intestinal dysfunction via inhibition of oxidative stress and the inflammatory response.

Nrf2 and NF-κB are closely involved in the oxidative stress (Gerstgrasser et al., 2016) and inflammatory (Wang et al., 2011) responses, respectively. Our previous research has demonstrated that ISL has anti-inflammatory and antioxidant effects in SAP, and that the underlying mechanisms for these effects are poorly elucidated. ISL has also been shown to have a protective effect in cerebral ischemia (Zeng et al., 2017) and in sepsis (Liu Q. et al., 2017) via inhibition of NF-κB activation. Oxidative stress associated with Nrf2 has been shown to be regulated by ISL (Wang et al., 2014). In SAP, we determined that ISL treatment inhibits activation of NF-κB and increases expression of the Nrf2 protein in the pancreas and intestines. Curiously, our results also showed that release of the inflammatory cytokines, IL-6 and TNF-α, in Nrf2^-/-^ mice was significantly greater than in WT mice, and that WT mice had a superior reduction in cytokines with ISL than the Nrf2^-/-^ mice. Mouse models of ischemia/reperfusion injury (Dai et al., 2018) and traumatic brain injury (Jin et al., 2008b) have suggested that NF-κB associated with the inflammatory response can be inhibited by regulating Nrf2 expression. Therefore, we assessed NF-κB protein expression in the pancreas and intestine following cerulein plus LPS treatment of Nrf2^-/-^ mice. Here, cerulein plus LPS was found to induce the expression of NF-κB in Nrf2^-/-^ mice significantly more than in WT mice. The effect of ISL inhibition on NF-κB activity in Nrf2^-/-^ mice was significantly reduced. For the first time, we found that ISL treatment inhibits the inflammatory response, at least in part, by regulating the Nrf2/NFκB pathways in mouse model of SAP.

One of the important pathologic processes of SAP is cell apoptosis (Athwal et al., 2014). Therefore, reducing apoptosis has been identified as a therapeutic target to prevent SAP progression. Increasing evidence shows that elevated expression of Nrf2 can inhibit apoptosis (Jung et al., 2014; Hu et al., 2017), and that ISL inhibits apoptosis in the model of cell injury (Zhao et al., 2015; Mahalingam et al., 2016; Hou et al., 2017). Moreover, ISL has been shown to exert protective effects by suppressing cell apoptosis in the digestive system (Redondo-Blanco et al., 2017). Based on the above research, it is reasonable to infer that ISL treatment may reduce apoptosis in SAP. In this study, we found that ISL reduced the number of apoptotic cells in intestinal and pancreatic tissue in the mouse model of SAP. The degree of histological injury and the number of apoptotic cells in Nrf2^-/-^ mice was greater than in WT mice after treatment with cerulein plus LPS, and the protective effects of ISL were weakened in Nrf2^-/-^ mice. Collectively, these findings suggest that Nrf2 deletion aggravates SAP-induced injury and that the protective effect of ISL on SAP is, at least partially, via regulation of Nrf2 protein.

One of the significant complications of SAP is intestinal dysfunction (Smit et al., 2016; Kang et al., 2017), which is when the intestinal barrier is destroyed allowing organisms to enter the bloodstream through the broken intestinal barrier (Dumnicka et al., 2017; Manohar et al., 2017). This can lead to multiple organ failure through sepsis. Therefore, maintaining intestinal integrity reduces morbidity associated with SAP and benefits recovery. Previous reports have indicated that ISL can help maintain integrity of the blood–brain barrier in the ischemia/reperfusion injury model (Zeng et al., 2017). There is also evidence that ISL exerts chondroprotective effects by maintaining the integrity of the cartilaginous mucosa in a mouse model of anterior cruciate ligament transection (Ji et al., 2017). ISL has also been reported to target GRP78 to chemosensitize breast cancer stem cells via β-catenin/ABCG2 signaling (Wang et al., 2014). As none of these animal studies considered TJ and AJ proteins there was insufficient evidence to consider trialing ISL to repair the intestinal barrier in humans. Therefore, we investigated the effects of ISL on intestinal barrier integrity in a mouse model of SAP. Our data showed that the decrease in intestinal TJ (P120-catenin) and AJ (occludin) proteins after SAP was inhibited by ISL treatment. This proves that ISL acts to repair the intestinal barrier in mice after SAP. Interestingly, other studies have shown that targeting Nrf2 can improve skin barrier function and photoprotection (Rojo de la Vega et al., 2017). We found that the expression of P120-catenin and occludin proteins in the intestines of Nrf2^-/-^ mice was much lower than in WT mice, and that ISL treatment could not significantly improve the P120-catenin and occludin proteins in Nrf2 ^-/-^ mice compared with WT mice. Collectively, these findings suggest that Nrf2 has a protective effect on the intestinal barrier, and that ISL maintains intestinal integrity by regulating the Nrf2 protein in a mouse of SAP.

## Conclusion

**FIGURE 7:**
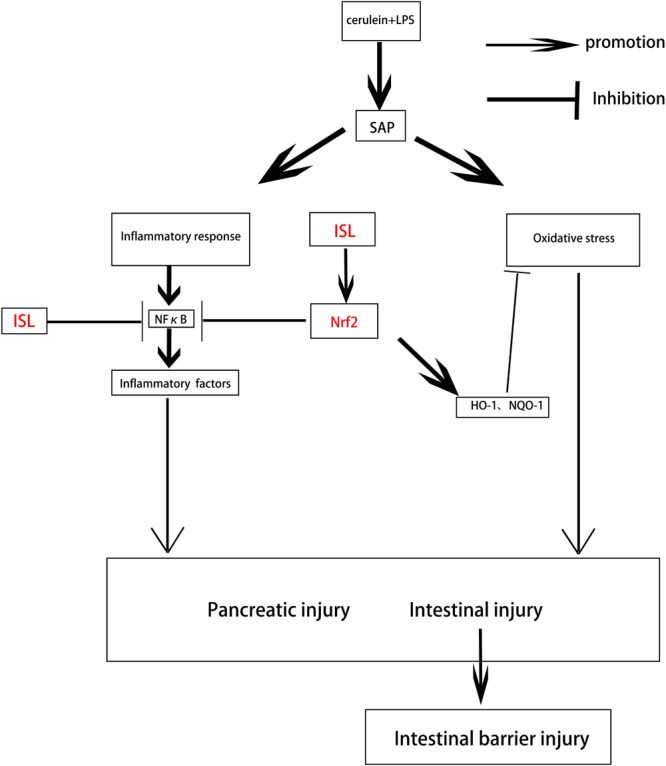
Diagram depicting the interaction between ISL and Nrf2 that results in protection against pancreatic injury and intestinal dysfunction after SAP. SAP induced by cerulein combined with LPS, which produces oxidative stress and an inflammatory response, leads to pancreatic injury and intestinal disorders. ISL inhibits NF-κB activation through the Nrf2/NF-κB pathway to inhibit inflammation. ISL also activates Nrf2 to inhibit oxidative stress and maintain the integrity of the intestinal barrier thereby reducing the injury associated with SAP.

We showed that ISL significantly reduced pancreatic and intestinal damage, and repaired intestinal barrier function by attenuating the oxidative stress and inflammatory response in a mouse model of SAP. Mice deficient in Nrf2 were more sensitive to the damage caused by oxidative stress and the inflammatory response in SAP. Furthermore, activation of NF-κB was closely related to Nrf2, with ISL reducing the inflammatory response in SAP via the Nrf2/NF-κB pathway. We have demonstrated that the Nrf2 protein repairs the intestinal barrier and that ISL maintains intestinal integrity via regulation of Nrf2 protein (**Figure 7**). These findings may serve as the basis for development of therapeutic strategies using ISL that could assist with recovery from SAP in clinical practice.

## Ethics Statement

Animal study protocols were approved by the Wenzhou Medical University Animal Policy and Welfare Committee (Approval Document No. wydw2014-0058).

## Author Contributions

MZ designed and carried out the work. Y-QW analyzed the experimental results. LX collected the data. JW analyzed and interpreted the data. KX drafted the article. JX critically revised the article. D-QC approved the final version of the article to be published.

## Conflict of Interest Statement

The authors declare that the research was conducted in the absence of any commercial or financial relationships that could be construed as a potential conflict of interest.
