# Corrigendum: Isoliquiritigenin Protects Against Pancreatic Injury and Intestinal Dysfunction After Severe Acute Pancreatitis *via* Nrf2 Signaling

**DOI:** 10.3389/fphar.2019.00788

**Published:** 2019-07-12

**Authors:** Man Zhang, Yan-Qing Wu, Ling Xie, Jiang Wu, Ke Xu, Jian Xiao, Da-Qing Chen

**Affiliations:** ^1^Department of Emergency, The Second Affiliated Hospital and Yuying Children’s Hospital of Wenzhou Medical University, Wenzhou Medical University, Wenzhou, China; ^2^Molecular Pharmacology Research Center, School of Pharmaceutical Science, Wenzhou Medical University, Wenzhou, China; ^3^Wenzhou University College of Life and Environmental Science, Wenzhou, China

**Keywords:** severe acute pancreatitis, intestinal dysfunction, isoliquiritigenin, Nrf2^-/-^, NF-κB, oxidative stress

In the original article, there was a mistake in **Figure 1**, **Figure 4G**, **Figure 5E** and **Figure 6E** as published. During the process of making substantial amendments to our manuscript, **Figures 1G, H** were replaced by **Figures 4D**, **E** by mistake. In **Figure 4G** and **Figure 5E**, the internal reference proteins, GAPDH were incorrect. The Sham group in **Figure 6E** was selected from another group by mistake. The corrected figures appear below.

**Figure 1:**
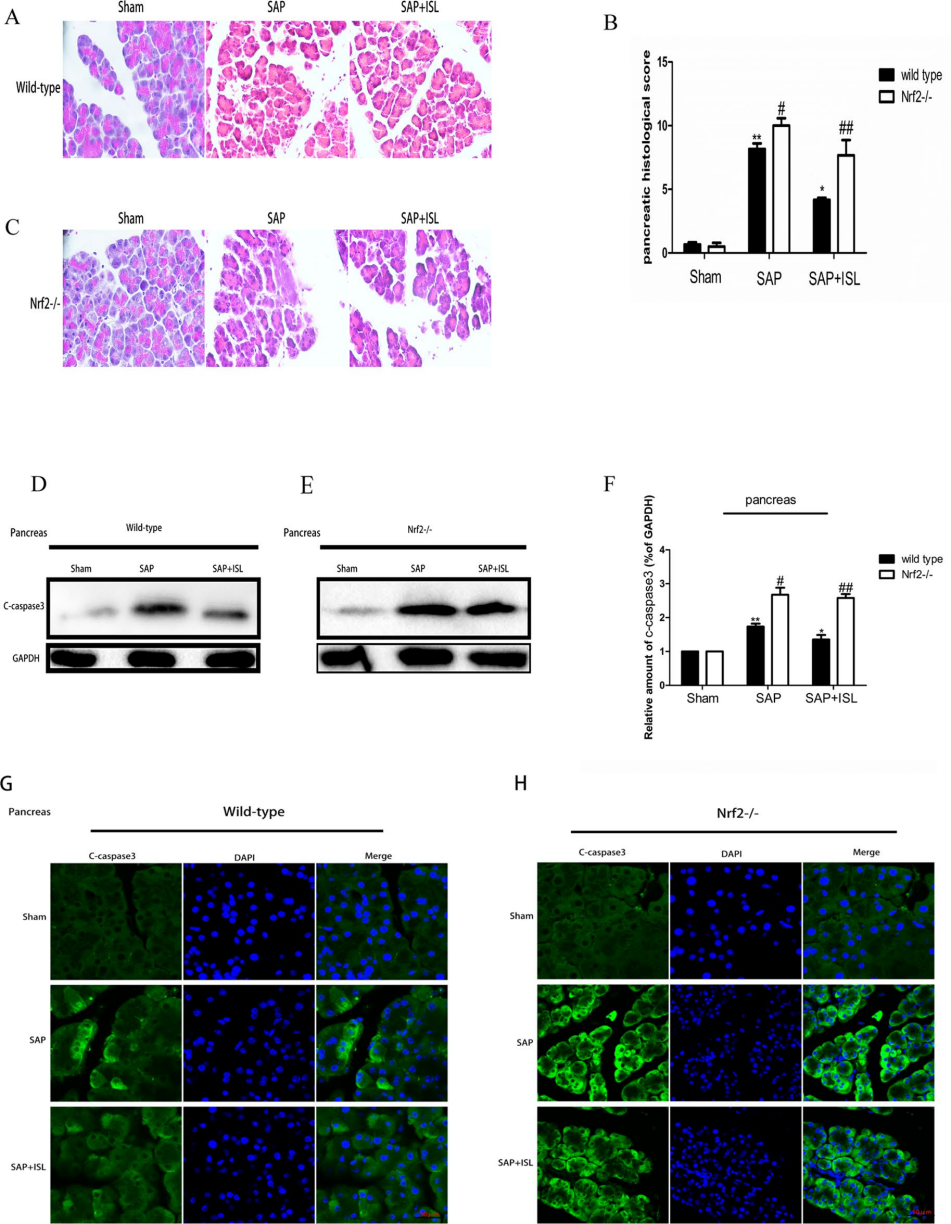
Isoliquiritigenin (ISL) treatment protects against combined cerulein plus LPS-induced severe acute pancreatitis (SAP) in pancreatic tissue. **(A**, **C)** Pancreatic morphological changes in the different groups at 24 h after SAP. **(B)** Pancreatic histological scores. **(D**, **E)** Expression of c-caspase-3 protein in pancreatic tissue from the different groups at 24 h after SAP. GAPDH was used as the loading control and for band density normalization. **(F)** Statistical graph of c-caspase-3 proteins. **(G**, **H)** Immunofluorescence staining for c-caspase-3 (green) and DAPI (blue) in the different groups. Results are expressed as mean ± SEM. n = 5 per group. *P < 0.05 and **P < 0.01 when comparison was made in WT mice. ^#^P < 0.05 and ^##^P < 0.01 when comparison was made between WT mice and Nrf2−/− mice.

**Figure 4:**
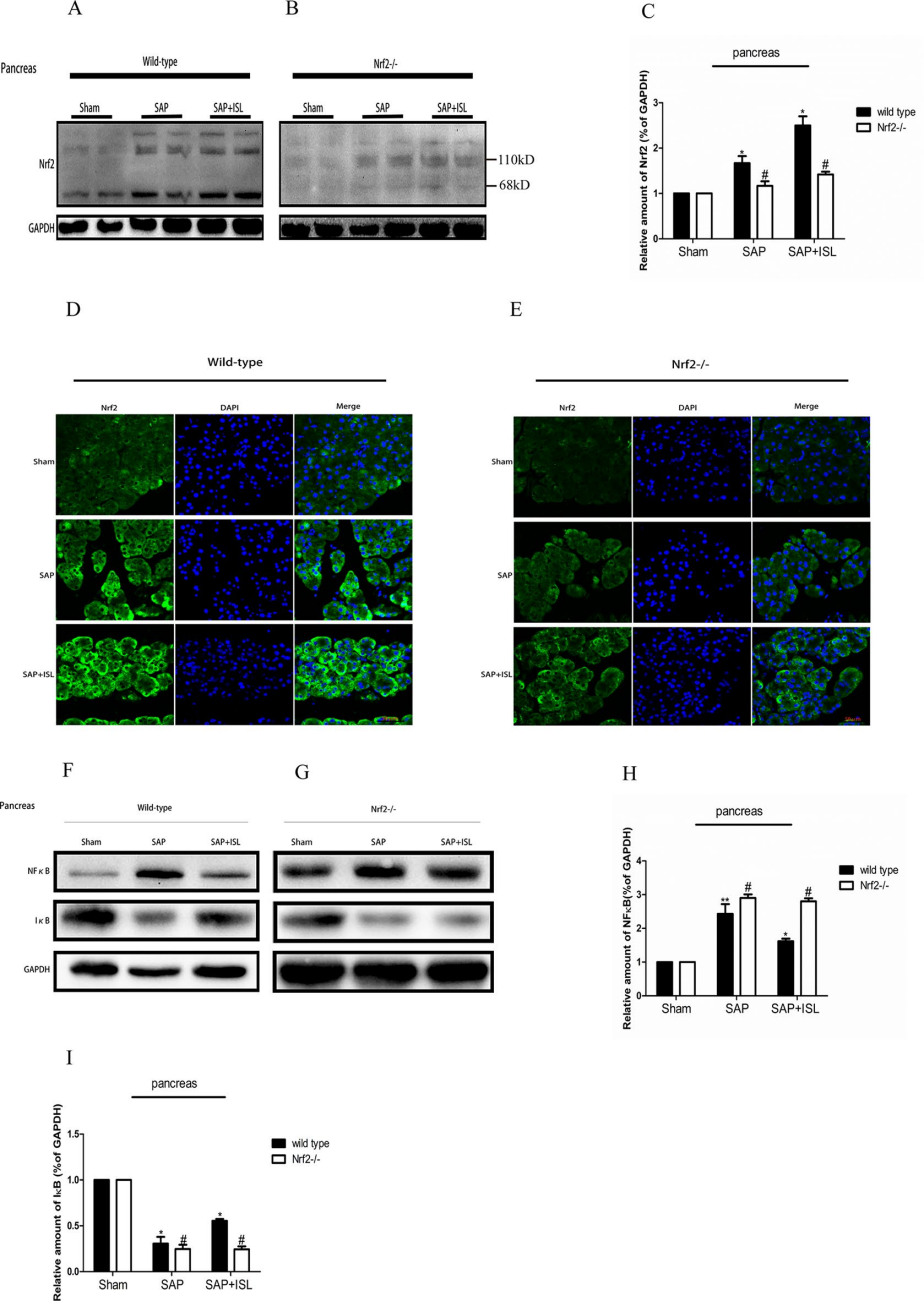
Effect of ISL treatment on NF-κB and Nrf2 in pancreatic tissue in WT and Nrf2−/− mice after SAP. **(A**, **B)** Protein expression of Nrf2 in pancreatic tissue of the different groups after SAP. GAPDH was used as the loading control and for band density normalization. **(C)** Statistical graph of Nrf2 and GAPDH protein in the different groups. **(D**, **E)** Immunofluorescence staining for Nrf2 (green) and DAPI (blue) in the different groups. **(F**, **G)** Protein expression of NF-κB and IκB in pancreatic tissue in the different groups after SAP. GAPDH was used as the loading control and for band density normalization. **(H**, **I)** Statistical graph of NF-κB, IκB, and GAPDH protein in the different groups. Results are expressed as mean ± SEM. n = 5 per group. *P < 0.05, **P < 0.01, and ***P < 0.001 when comparison was made in WT mice. ^#^P < 0.05 when comparison was made between WT mice and Nrf2−/− mice.

**Figure 5:**
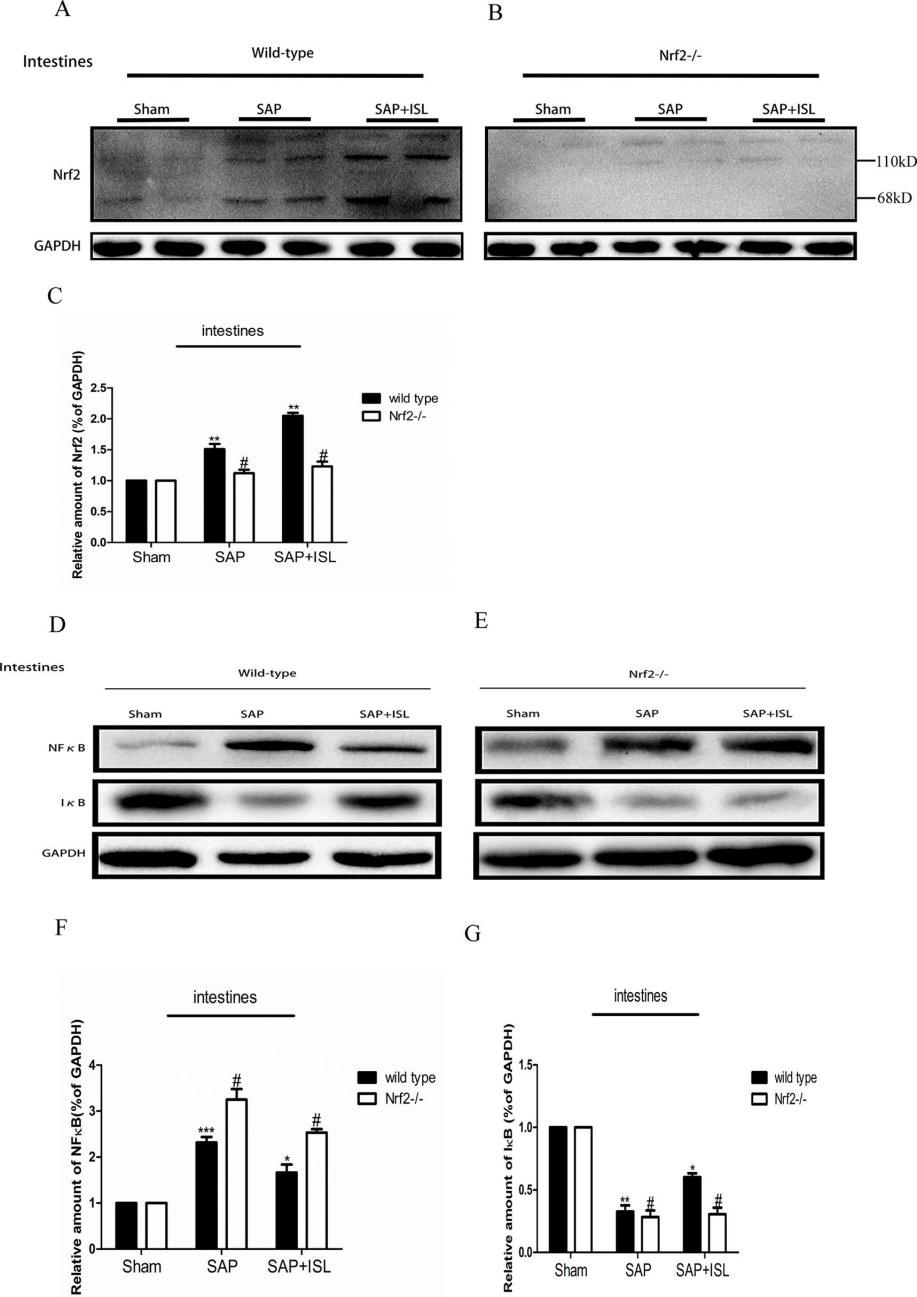
Effect of ISL treatment on NF-κB and Nrf2 in intestinal tissue in WT and Nrf2−/− mice after SAP. **(A**, **B)** Protein expression of Nrf2 in intestinal tissue in the different groups after SAP. GAPDH was used as the loading control and for band density normalization. **(C)** Statistical graph of Nrf2 and GAPDH protein in the different groups. **(D**, **E)** Protein expression of NF-κB and IκB in pancreatic tissue in the different groups after SAP. GAPDH was used as the loading control and for band density normalization. **(F**, **G)** Statistical graph of NF-κB and IκB. GAPDH protein in the different groups. Results are expressed as mean ± SEM. n = 5 per group. *P < 0.05, **P < 0.01, and ***P < 0.001 when comparison was made in WT mice. ^#^P < 0.05 when comparison was made between WT mice and Nrf2−/− mice.

**Figure 6:**
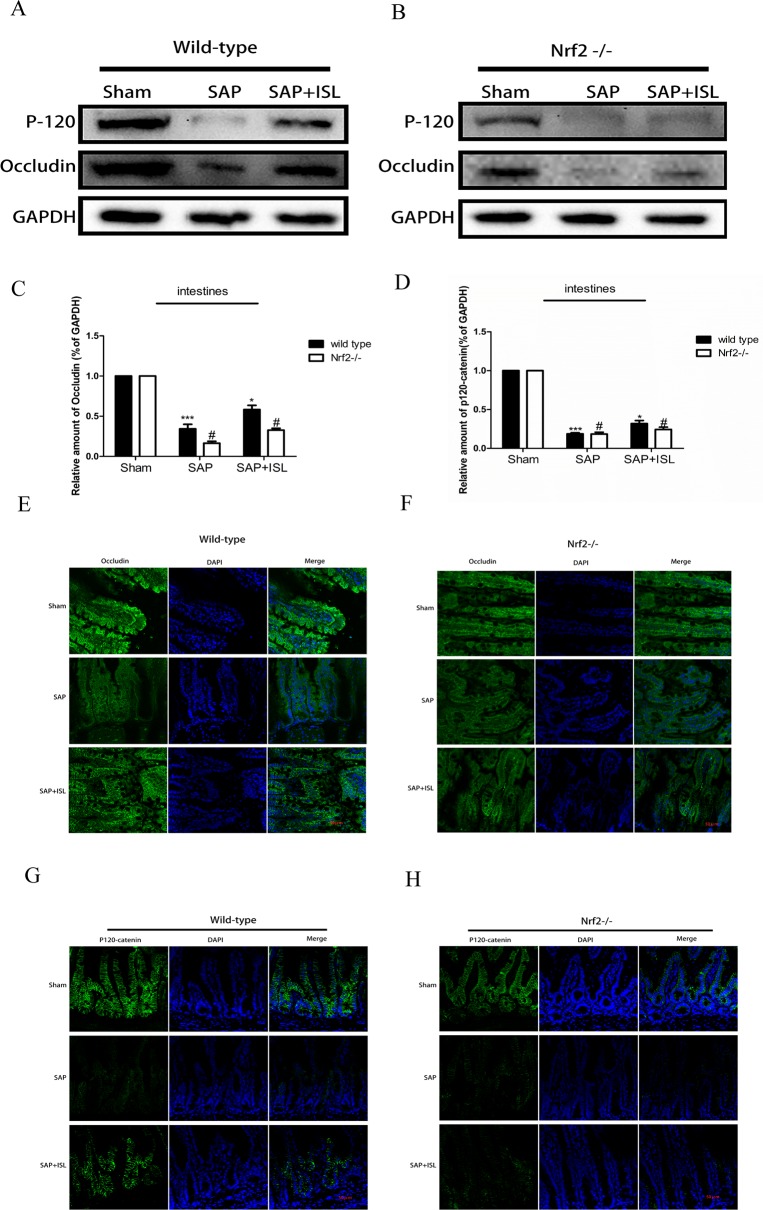
Effect of Nrf2 deletion on maintenance of intestinal barrier integrity after SAP. **(A**, **B)** Protein expression of P-120-catenin and occludin in pancreatic and intestinal tissue in the different groups after SAP. GAPDH was used as the loading control and for band density normalization. **(C**, **D)** Statistical graph of P-120-catenin, occludin, and GAPDH protein in the different groups. **(E–H)** Immunofluorescence staining for P-120-catenin (green), occludin (green), and DAPI (blue) in the different groups. Results are expressed as mean ± SEM. n = 5 per group. *P < 0.05 and ***P < 0.001 when comparison was made in WT mice. ^#^P < 0.05 when comparison was made between WT mice and Nrf2−/− mice.

The authors apologize for this error and state that this does not change the scientific conclusions of the article in any way. The original article has been updated.

